# Synthesis and Characterization of Supermagnetic Nanocomposites Coated with Pluronic F127 as a Contrast Agent for Biomedical Applications

**DOI:** 10.3390/pharmaceutics15030740

**Published:** 2023-02-23

**Authors:** Maria Janina Carrera Espinoza, Kuen-Song Lin, Meng-Tzu Weng, Sikhumbuzo Charles Kunene, You-Sheng Lin, Chun-Ming Wu

**Affiliations:** 1 Department of Chemical Engineering and Materials Science, Yuan Ze University, Chung–Li District, Taoyuan City 320, Taiwan; 2 Department of Internal Medicine, National Taiwan University Hospital, Taipei 100, Taiwan; 3 Department of Medical Research, National Taiwan University Hospital Hsin-Chu Branch, Hsinchu 302, Taiwan; 4 National Synchrotron Radiation Research Center, Hsinchu Science Park, Hsinchu 300, Taiwan

**Keywords:** supermagnetic nanocomposites, Pluronic F127, in vitro test, liver cancer, doxorubicin, HepG2 cell line, drug delivery

## Abstract

Nanomedicine has garnered significant interest owing to advances in drug delivery, effectively demonstrated in the treatment of certain diseases. Here, smart supermagnetic nanocomposites based on iron oxide nanoparticles (MNPs) coated with Pluronic F127 (F127) were developed for the delivery of doxorubicin (DOX) to tumor tissues. The XRD patterns for all samples revealed peaks consistent with Fe_3_O_4_, as shown by their indices (220), (311), (400), (422), (511), and (440), demonstrating that the structure of Fe_3_O_4_ did not change after the coating process. After loading with DOX, the as-prepared smart nanocomposites demonstrated drug-loading efficiency and drug-loading capacity percentages of 45 ± 0.10 and 17 ± 0.58% for MNP-F127-2-DOX and 65 ± 0.12 and 13 ± 0.79% for MNP-F127-3-DOX, respectively. Moreover, a better DOX release rate was observed under acidic conditions, which may be credited to the pH sensitivity of the polymer. In vitro analysis demonstrated the survival rate of approximately 90% in HepG2 cells treated with PBS and MNP-F127-3 nanocomposites. Furthermore, after treatment with MNP-F127-3-DOX, the survival rate decreased, confirming cellular inhibition. Hence, the synthesized smart nanocomposites showed great promise for drug delivery in liver cancer treatment, overcoming the limitations of traditional therapies.

## 1. Introduction

Cancer is one of the fatal diseases responsible for more than eight million deaths per year globally [1]. Different types of cancers, such as breast, colorectal, liver, and lung cancers, are the most commonly diagnosed cancers in women in Asia and Europe. Interestingly, liver and lung cancers remain the leading causes of mortality worldwide [2,3]. The liver is a vital organ that regulates most of the chemical processes in the blood and is responsible for eliminating a substance called bile from the body, which helps eliminate waste products. This vital organ is also responsible for metabolizing and breaking down medications in a manner that is safe for utilization in the body [4]. Several studies have demonstrated that hepatocellular carcinoma (HCC), commonly known as liver cancer, is one of the top five causes of death in 90 countries globally. It is predicted that the cases and deaths will rise over the coming years as the world’s population grows [5,6]. This fatal disease is mainly associated with several causes, such as infections by B or C hepatitis virus (HBV and HCV), alcohol consumption, obesity, smoking, cirrhosis, diabetes, iron overload, and nonalcoholic fatty liver disease, among other different causes [7,8,9].

Currently, surgical intervention, radiation, and chemotherapy are the main approaches used for cancer treatment. However, these therapies have some disadvantages and side effects. Radiotherapy has several drawbacks attributed to long-term exposure to high doses of ionizing radiation (X-rays), which can induce cellular changes and alterations [10,11]. Additionally, owing to the lack of cancer symptoms, surgical intervention is often performed when the disease is already advanced (type III or IV), and in most cases, it can cause collateral damage to the patient, such as metastasis. Moreover, chemotherapy aims to treat patients with intravenous drug administration, leading to widespread systemic distribution. This treatment is not specific because it provokes therapeutic drugs to attack not only cancerous but also healthy cells, generating side effects, such as anemia, hair loss, bruising, bleeding, sore mouth, loss of appetite, and others [12,13]. Furthermore, widespread distribution and rapid elimination to organs and different tissues require the administration of a large amount of a drug, which causes toxicity and worsens the quality of life of patients [14]. Hence, to prevent all of these drawbacks as well as drug resistance, it is crucial to develop a smart nanocomposite with targeting ability to successfully treat HCC to avoid spreading drugs to organs and tissues that are not the target sites.

**Scheme 1:**
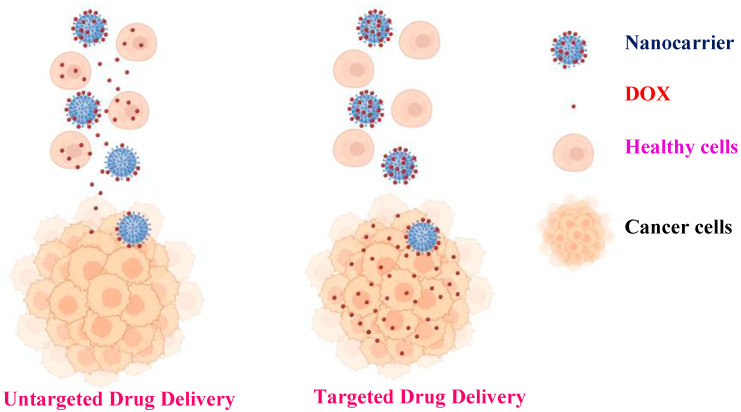
Comparison between untargeted and targeted drug delivery.

Advances in nanomedicine have demonstrated that the use of targeted nanomaterials along with stimuli-responsive polymers can improve the efficacy of cancer treatment via enhancement of the clinical indices of the active compounds engineered within the nanocomposites [15,16,17,18]. The use of targeted nanoparticles increases the possibility of reducing the side effects of antitumor drugs by administrating lower but more precisely targeted doses to specific tissues (Scheme 1) [19]. Magnetic nanoparticles (MNPs) have been studied and applied in biomedicine owing to their physical and chemical properties, such as good solubility, high drug-loading capacity, magnetic hyperthermia, magnetic resonance imaging (MRI), excellent biocompatibility, and biodegradability [20]. The application of magnetic nanoparticles offers several benefits for drug delivery. In general, they can target precise sites in the body, thus decreasing the systemic distribution of cytotoxic complexes and increasing uptake at the target tissue, resulting in potent therapeutic effects at lower doses [21]. The biomedical applications of MNPs, in addition to biomedicine, have generated a great impact on the diagnosis of several diseases, such as cancer and infectious diseases. Additionally, the combined use of biomedicine and nanomedicine has created a platform called theranostics (therapeutics and diagnostics). Significant progress has been made in nanoparticle engineering in recent years, as well as innovative new hybrids for multifunctional modalities, such as imaging, biosensing, chemotherapeutic or photothermal agents, and antimicrobials [22]. 

The incorporation of polymers onto the surface of the nanoparticles facilitates drug loading via surface adsorption, entrapping, or encapsulation [23,24,25]. Moreover, the drug-loading ability and biomedical properties of MNPs attributed to various surface coatings are the factors that can aid in avoiding toxicity. Several organic and inorganic compounds have been used as coating materials for the surface modification of nanocargoes to reduce their toxicity and increase their loading ability. Stimuli-sensitive nanocomposites that react to pH, temperature, and magnetic fields have garnered significant interest owing to their ability with respect to constant and controlled release to particular tissues [26]. pH-sensitive nanoparticles have developed rapidly in the field of biomedicine because of their ability to induce drug release at lower pH due to ionizable functional groups on the polymer backbone or side chain, which facilitates the dissolution of the carriers. These smart nanocomposites can be used to target tumor tissues, lysosomes, and endosomes, where the pH is relatively low [27,28,29]. Pluronic F127 is one of the most common smart copolymers used for drug delivery owing to its pH sensitivity [30,31,32]. It is a copolymer composed of poly-ethylene oxide (PEO) and hydrophobic poly-propylene oxide (PPO), with an ABA triblock arrangement (PEO–PPO–PEO) [33,34,35]. The advantage of using this copolymer is the PEO outer crown, which confers antifouling properties to prevent nanocomposite aggregation, protein adsorption, and recognition by the reticuloendothelial system (RES). Furthermore, the hydrophobic PPO core can be adjusted to encapsulate hydrophobic anticancer drugs [36,37,38,39]. This non-toxic copolymer has been applied in different pharmaceutical areas, including tissue engineering, the development of drug carriers, and gene delivery [40,41,42,43]. 

Doxorubicin (DOX) is a common cancer drug with high antitumor activity and strong side effects, such as cardiotoxicity [44]. The application of DOX has demonstrated positive results for the treatment of several types of cancers, including breast, lung, gastric, liver, skin, bone, ovarian, and thyroid cancers [45,46]. DOX mediates the generation of oxygen-containing reactive species (ROS) in different tumor cells. However, the specific function of ROS in the DOX-mediated killing of tumor cells remains unclear. Additionally, the intracellular oxidative stress generated by DOX is typically attributed to the overexpression of antioxidant enzymes, which can prevent apoptosis in cancer cells. However, the lack of endogenous antioxidants could cause cancer cells to be more susceptible to apoptosis. In cardiomyocytes, the target organelles of DOX toxicity are the mitochondria, where DOX accumulates over time, resulting in approximately two- to three-fold magnitude higher DOX concentration than its extracellular concentration in culture [47,48]. The development of an innovative carrier to entrap DOX into the polymer may decrease side effects and augment the quality of life of patients. The high payload of DOX is responsible for its sustained release behavior and good cellular internalization capability during release, rendering this nanocomposite suitable for drug delivery. The present study aimed to develop an innovative smart nanocomposite based on a pH-sensitive smart polymer coated on the surface of MNPs loaded with the anticancer drug DOX (MNP-F127-DOX) for the treatment of liver cancer. Although researchers have studied the application of MNP-F127-DOX against breast cancer and oral epithelial cancer cells (MCF-7 and C152, respectively), few studies have explored its inhibitory ability against liver cancer cells (HepG2) [49]. This smart nanocomposite presented remarkable aqueous colloidal stability, magnetic response capacity, and the ability to load antitumor drugs for constant and low drug release, thereby circumventing side effects and providing a better quality of life for patients.

## 2. Materials and Methods

### 2.1. Materials, Chemicals, and Apparatus

Pluronic F127 (F127), iron (III) chloride (FeCl_3_–6H_2_O), hydrochloric acid (HCl), sodium hydroxide (NaOH), sodium acetate (NaCH_3_COO), sodium citrate (Na_3_C_6_H_5_O_7_), phosphate-buffered saline (PBS), ethylene glycol (MEG), and diethylene glycol (DEG) were obtained from Sigma Aldrich. Cancerous cells (HepG2), dimethyl sulfoxide (DMSO), Dulbecco’s modified Eagle medium (DMEM), doxorubicin (DOX), 3-(4,5-dimethyl thiazol-2-yl)-2,5-diphenyltetrazolium bromide (MTT), and DAPI solution were obtained from the National Taiwan University Hospital, Taipei, Taiwan.

### 2.2. Synthesis of the Nanocomposites

MNPs were synthesized as defined in our previous study [49]. Briefly, 2.4 g Na_3_C_6_H_5_O_7_, 16 g FeCl_3_–6H_2_O, and 16 g NaCH_3_COO were diluted in a solution of 320 mL MEG and 80 mL DEG. The solution was magnetically stirred for 3 h. Subsequently, the yellowish solution was heated in a Teflon-lined stainless-steel autoclave at 200 °C for 12 h. Then the black solution was washed several times with ethanol and deionized (DI) water to avoid contamination and subsequently dried at 65 °C for 12 h. Additionally, the surface-modified MNPs with F127 were formulated using two different concentrations of polymer (5 and 10%), mixed with 200 mg MNPs, and stirred overnight. The nanocomposites were washed numerous times with ethanol and DI water. Finally, the nanocomposites (MNP-F127-2 and MNP-F127-3) were dried at 65 °C overnight.

### 2.3. Characterization

The average particle size was studied using dynamic light scattering (DLS, HORIBA, Kyoto, Japan). Samples were diluted in ultrapure water to the appropriate concentration to avoid multiscattering. The measurement was completed at 25 °C and each parameter was measured in triplicate. The morphology and structure were obtained using field-emission scanning electron microscopy/energy-dispersive X-ray spectroscopy (FE-SEM/EDS, HITACHI, Hitachi, Japan) using solid samples, while for the transmission electron microscopy (TEM, HITACHI, Hitachi, Japan), one drop of the as-synthetized nanoparticles solution was separately placed on a copper grid coated with Formvar/carbon film and dried under vacuum for 10 h. In addition, the functional groups of the studied nanocomposites were detected by Fourier transform infrared (FTIR, PerkinElmer, Waltham, MA, USA); the spectra were the average of 50 scans recorded at a resolution of 4 cm^−^^1^ in a range of 4000 to 500 cm^−^^1^. Moreover, the crystalline structures were detected using X-ray diffraction (XRD, MXP18, Mac Science, Japan) with Cu-Kα radiation in the range of 2θ = 20–80°. Furthermore, the chemical compositions of the nanocomposites were determined by X-ray photoelectron spectroscopy (XPS; Fison-ESCA, Tokyo, Japan) calibrated to a carbon peak (C 1s). Finally, the local structures and dynamic properties of the nanocomposites were detected by electron paramagnetic resonance spectrometry (EPR; JEOL, Akishima, Japan).

### 2.4. Formulation of MNP-F127-DOX Core-Shell Nanocomposites

The MNP-F127-2 and MNP-F127-3 were loaded with DOX following our previous study [50]. Briefly, 3 mg each of MNP-F127-2 and MNP-F127-3 was separately mixed with 1 mg of DOX and 1 mL of DI water for 48 h under constant shaking in the dark. Subsequently, the formulations were centrifuged at 65,000 rpm for 20 min, and the acquired pellet was oven-dried at 65 °C overnight. The drug-loading efficiency (DLE) and drug-loading capacity (DLC) were measured as follows:

(1) $$DLE\;\left(\%\right)=\frac{M_{t}\;}{M_{o}}\times 100$$

(2) $$DLC\;\left(\%\right)=\frac{M_{t}\;}{M_{t}+M_{L}}\times 100$$

where *M_o_*, *M_L_*, and *M_t_* denote the primary drug content, total quantity of the nanocomposite, and the mass of the encapsulated drug, correspondingly.

### 2.5. In Vitro DOX Release under Conditions Involving Various pH 

The drug release for MNP-F127-3-DOX was performed under different conditions (pH 7.4 and 5.4). Briefly, 2 mg MNP-F127-3-DOX was diluted in 4 mL PBS and subsequently placed into a dialysis bag (MWCO: 3500). The assembled dialysis bags were placed separately into 18 mL PBS in various pH conditions. The release experiment was completed in the dark at 37 °C with continuous stirring for 72 h. At specific intervals, 5 mL of the mixture was collected and measured. The solution was then transferred back to the initial suspension. UV-Vis (483 nm) spectroscopy was used to estimate the concentration of DOX released into the solution. The formula used to measure the percentage of DOX released is as follows:

(3) $$Drug\;release\;\left(\%\right)=\frac{DOX\;in\;dialysis\;medium\;}{Total\;DOX\;in\;the\;system}\times 100$$

### 2.6. Cytotoxicity Assay

The cytotoxicity assay was performed using human liver carcinoma cell lines (HepG2), following our previous studies [49,50]. Briefly, 1 × 10^6^ cells were seeded in 96-well plates (n = 3) and incubated for 24 h. DMEM was replaced with fresh medium containing increasing concentrations of PBS (control), MNP-F127-3, MNP-F127-3-DOX, and free DOX. The treated cells were incubated at 37 °C for 24, 48, and 72 h. After that, 20 µL of MTT solution was injected into each well and incubated for 4 h at 37 °C. Subsequently, DMEM was replaced with DMSO and incubated for 15 min. Finally, the absorbance was measured at 483 nm; the final data are a comparison of untreated control cells to the percentage of viable cells.

### 2.7. Cellular Uptake

The cellular uptake of PBS (control), MNP-F127-3, MNP-F127-3-DOX, and free DOX was observed using fluorescence microscopy, as previously reported [49,50,51,52,53,54,55,56]. Briefly, a piece of coverslip was placed into each well of 6-well plates, and HepG2 cancerous cells were seeded on top at a cell density of 5 × 10^3^ cells/well. After 24 h of incubation at 37 °C, the HepG2 cells were treated with PBS (control), MNP-F127, MNP-F127-DOX, and free DOX at equivalent concentrations of 2 μL, and incubated at similar conditions. After this period, the medium was discarded, and the coverslip containing the HepG2 cancerous cells was washed with fresh PBS and fixed with 75% ice-cold ethanol for 20 min. Subsequently, the cells were washed with fresh PBS and treated with DAPI solution for 15 min. Finally, the coverslips containing the treated cells were rinsed with PBS and observed under a microscope for analysis. DAPI emission was detected at a wavelength of 480 nm.

### 2.8. Statistical Analyses

Analysis of variance (ANOVA) was applied for statistical analysis. Data were analyzed in triplicate and are presented as mean ± standard deviation (SD). The data obtained at * p < 0.05 were considered statistically significant.

## 3. Results and Discussion

### 3.1. Characterization of MNP and MNP-F127 

**Scheme 2:**
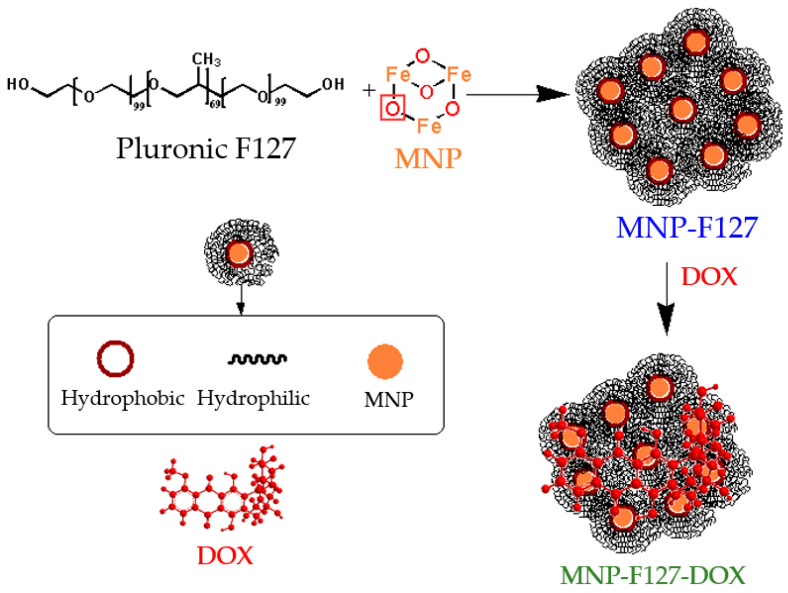
Schematic illustration of the chemical reactions of the as-synthetized nanocomposites.

The chemical interaction between the MNP and F127 was accomplished by hydrogen bonding. After conjugation, Pluronic F127 and DOX were functionalized via hydrophobic interactions due to the amphiphilic (hydrophobic and hydrophilic) property of the copolymer and the hydrophobic characteristic of DOX [57,58], as is shown in Scheme 2.

**Figure 1:**
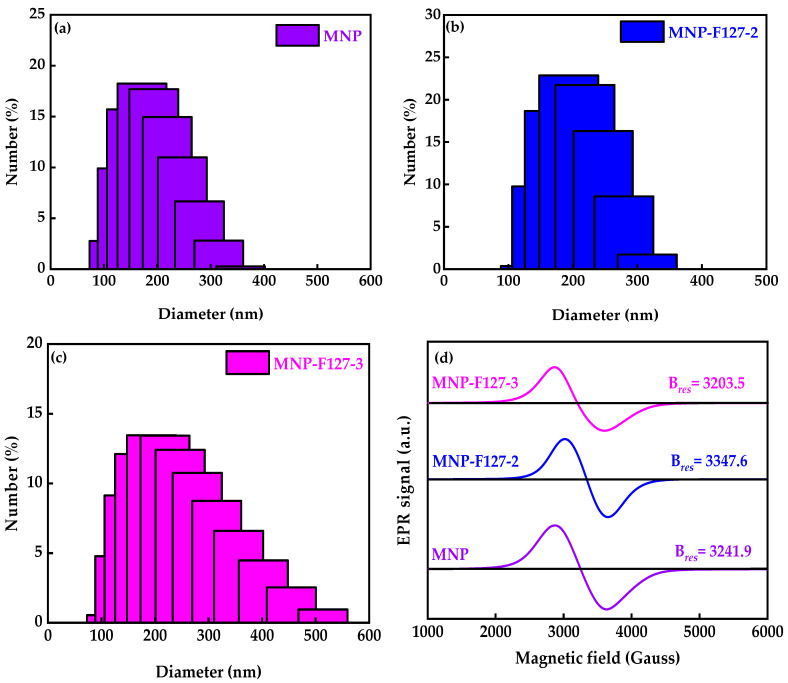
(**a**–**c**) Dynamic light scattering particle size distribution and (**d**) electron paramagnetic resonance of MNP, MNP-F127-2, and MNP-F127-3 nanocomposites.

To define the size distribution and colloidal stability of the as-synthesized smart nanocomposites, DLS analyses were performed. The average diameter and polydispersity index (PDI) of the MNPs were 168.9 nm and 0.298, respectively (Figure 1a). After coating with F127, the average diameter and PDI for MNP-F127-2 were 194.6 nm and 0.111, while for MNP-F127-3, they were 212.2 nm and 0.217, respectively (Figure 1b,c), confirming the polymeric coating on the surface of the MNPs. As the polymer concentration increased, the average diameter also increased, indicating an effective coating on the surface of the smart nanocomposites. EPR analyses were performed to determinate the magnetic characteristics at high frequency owing to the resonance originating from the interaction between electromagnetic waves and spins (Figure 1d). The data revealed strong and broad asymmetry resonance signals at a field of around 3203.5, 3347.6, and 3241.9 G for MNP, MNP-F127-2, and MNP-F127-3 nanocomposites, respectively. These line broadenings might arise from the dipolar interaction between MNPs [52,53]. These results can be attributed to spin disorder probably coming from the antiferromagnetic interaction between the neighboring spins in the MNPs. The *g* values for MNP, MNP-F127-2, and MNP-F127-3 were 2.16, 2.11, and 2.20, respectively, which ratifies the magnetic behavior of the nanocomposites [54,55,56].

**Figure 2:**
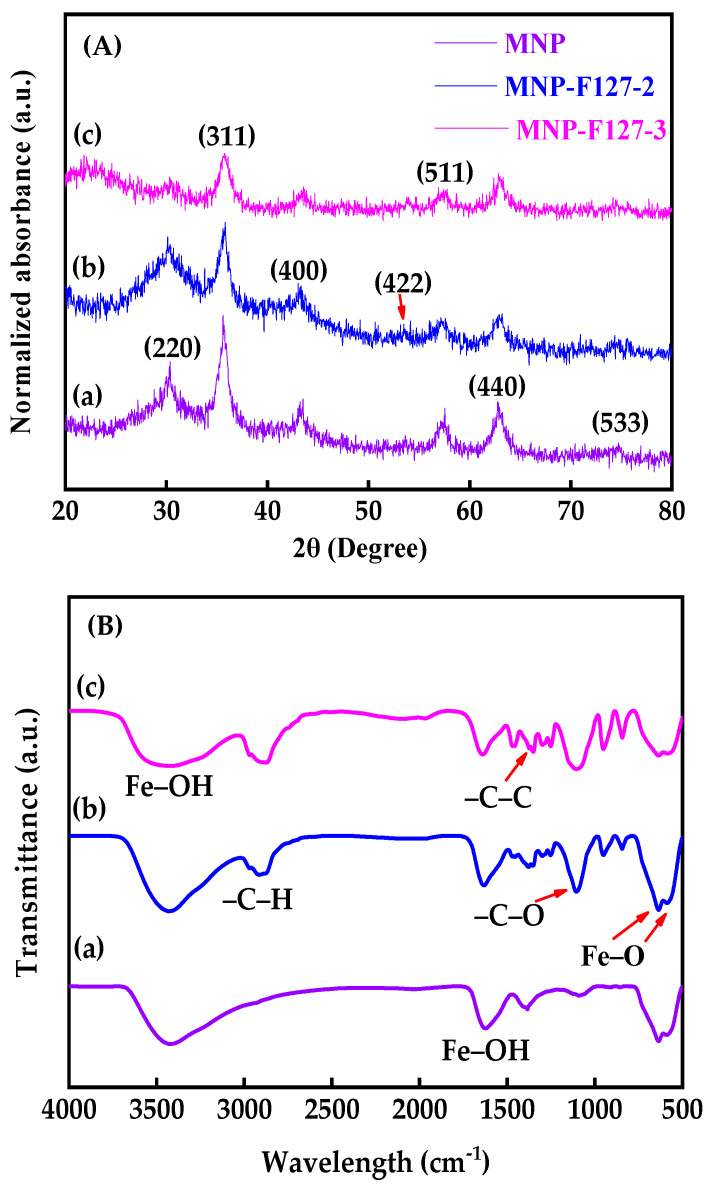
(**A**) XRD patterns and (**B**) FTIR absorbance spectra of (a) MNP, (b) MNP-F127-2, and (c) MNP-F127-3 nanocomposites.

The XRD patterns were conducted to investigate the structural parameters of the as-synthesized MNP, MNP-F127-2, and MNP-F127-3 smart nanocomposites. The XRD patterns showed diffraction peaks with 2θ values of 30.2°, 36.5°, 42.91°, 53.8°, 57.3°, 63.21°, and 73.78°, belonging to the (220), (311), (400), (422), (511), (440), and (533) planes of crystalline iron oxide nanoparticles, respectively (JCPDS No. 89–3854) [57,58,59]. This confirms that the Fe_3_O_4_ structure was retained during the coating process. Additionally, the broad nature of the diffraction peaks proposed that the as-synthetized nanocomposites present small particle sizes [60,61,62]. This result confirms the spinal state structure of the as-synthetized MNP, MNP-F127-2, and MNP-F127-3 smart nanocomposites (Figure 2A). Furthermore, FTIR spectra was performed to characterize the as-synthesized nanocomposites as well as to understand the existing surface functional groups in the metal interactions. The FTIR spectra of MNP, MNP-F127-2, and MNP-F127-3 nanocomposites showed strong bands at 579 and 635 cm^−1^, which belong to the vibration of the Fe–O bonds, confirming the formation of MNPs (Figure 2B).

**Figure 3:**
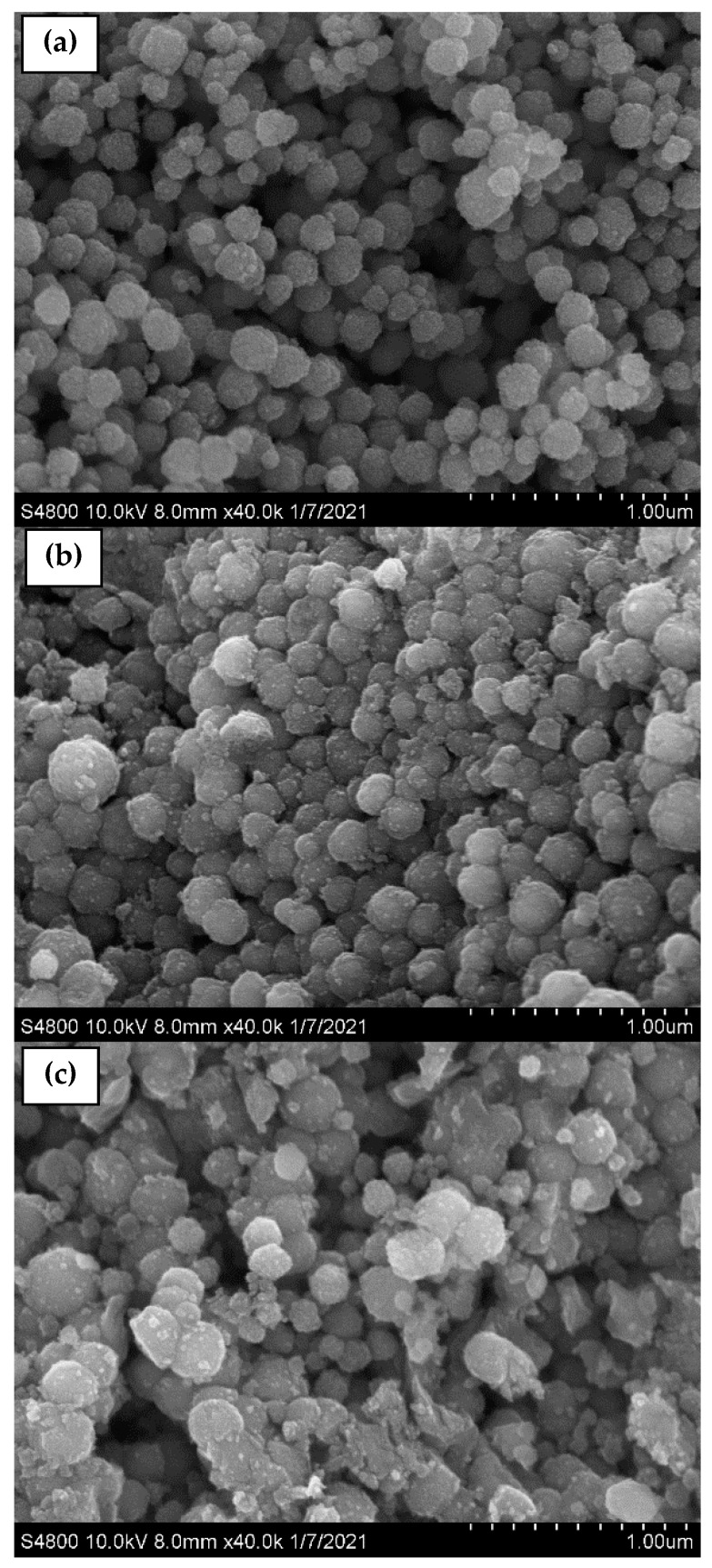
FE-SEM micrographs of as-synthesized (**a**) MNP, (**b**) MNP-F127-2, and (**c**) MNP-F127-3 nanocomposites.

Furthermore, the band at 592 cm^−1^ corresponds to the Fe–O stretching vibration of tetrahedral sites of the spinel structure. In addition, two bands of *ν*(Fe–OH) were recognized at 1609 and 3369 cm^−1^, corresponding to the stretching vibration of the hydroxyl groups on the surface of the MNPs [61,62,63,64]. After coating, the samples containing F127 presented peaks located at 2889, 1402, and 1110 cm^−1^ belonging to the asymmetric stretching vibrations –C–H, –C–C, and –C–O, respectively, of the polymer ascribed to the cross-linking of the hydrophilic part of Pluronic F127 and Fe^+^ cations [63,64]. The sizes and morphologies of the synthesized MNP, MNP-F127-2, and MNP-F127-3 nanocomposites were investigated using FE-SEM. The obtained images of the MNP, MNP-F127-2, and MNP-F127-3 nanocomposites are shown in Figure 3a–c. The data revealed quasi-spherical shapes with rough surfaces, and the particles were aggregated owing to their magnetic properties. After coating, the nanocomposites presented lower aggregation, slightly larger particle size, and a smoother surface, which may be due to the presence of F127 on the surface of the MNPs. In addition, the obtained nanocomposites were found to be increased in the range of 50–100 nm, due to the aggregation produced by their magnetic properties [64,65].

**Figure 4:**
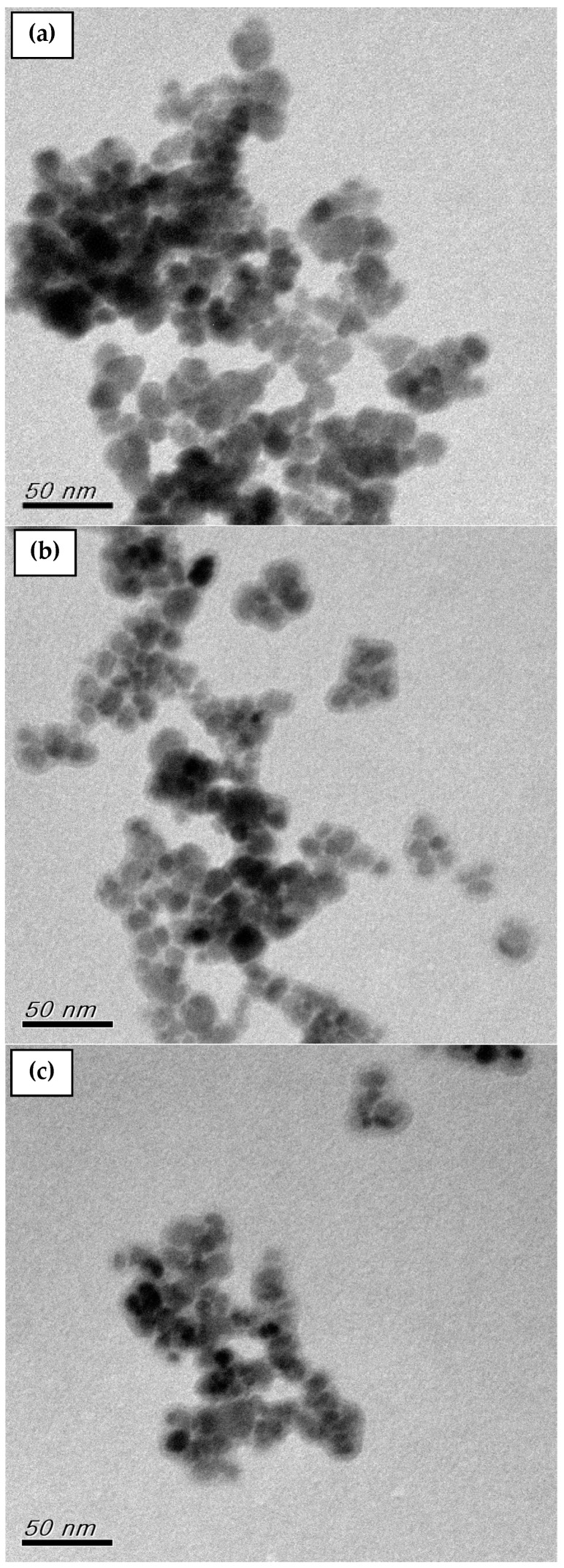
TEM images of as-synthesized (**a**) MNP, (**b**) MNP-F127-2, and (**c**) MNP-F127-3 nanocomposites.

Additionally, TEM was used to determine the structure and size of the MNP, MNP-F127-2, and MNP-F127-3 nanocomposites. The TEM micrographs of the nanocomposites are shown in Figure 4a–c. The micrograph for the MNPs reported aggregation between particles and varied sizes from 10 to 20 nm. After coating, the MNP-F127-2 and MNP-F127-3 nanocomposites reported a slight agglomeration and the diameter of the nanocomposites increased as a result of the coating layer of the F127 onto the surface of the MNPs [66,67,68].

### 3.2. DOX Encapsulation and Release 

**Figure 5:**
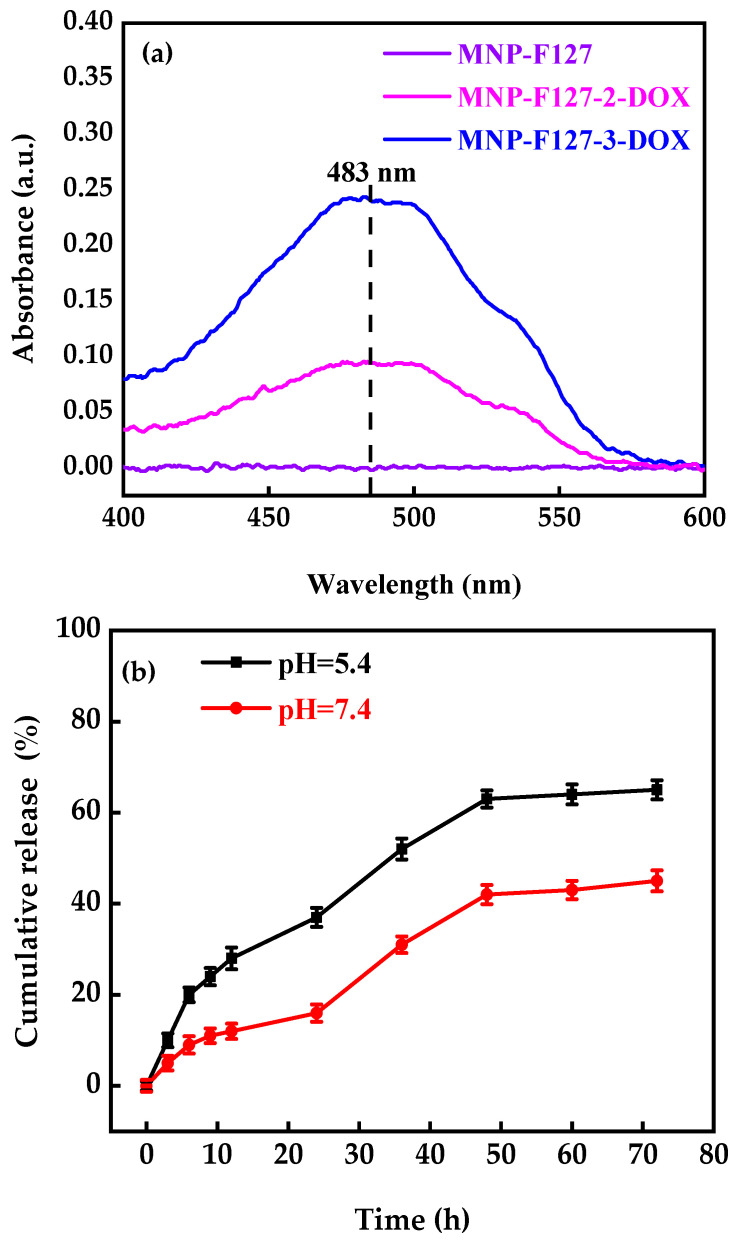
(**a**) UV-Vis spectra and (**b**) in vitro release profile of MNP-F127-3-DOX under different pH values (pH = 5.4 and 7.4) at 37 °C.

A UV-Vis spectrometer was used to determine the successful encapsulation of DOX (Figure 5). The MNP, MNP-F127-2-DOX, and MNP-F127-3-DOX nanocomposites were compared to free DOX. The results revealed a DOX-related absorbance peak at 483 nm associated with the MNP-F127-2-DOX and MNP-F127-3-DOX, confirming the successful DOX encapsulation. The DLE and DLC percentages of MNP-F127-2-DOX were 45 ± 0.10 and 17 ± 0.58%, correspondingly, whereas those of MNP-F127-3-DOX were 65 ± 0.12 and 13 ± 0.79%, respectively.

**Figure 6:**
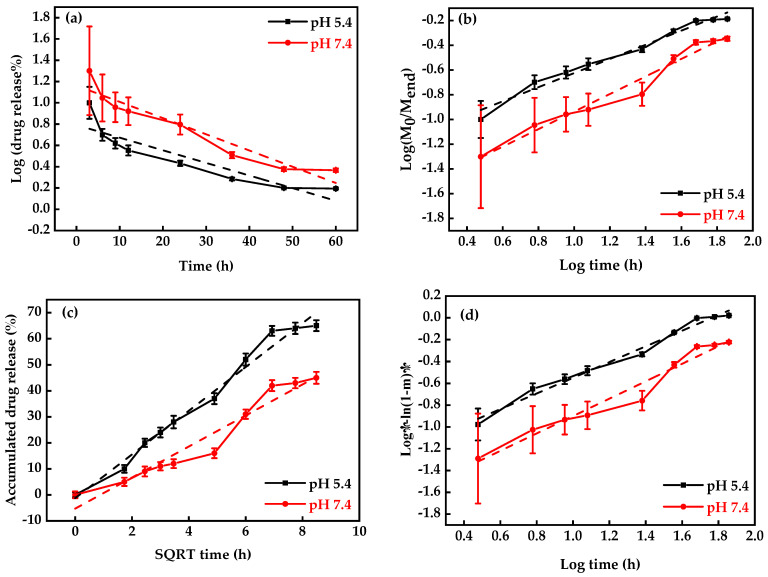
Drug-release kinetics plots: (**a**) Korsmeyer–Peppas, (**b**) First-order, (**c**) Weibull, and (**d**) Higuchi models under different pH values (pH = 5.4 and 7.4) at 37 °C (n = 3).

DOX-release profiles at different pH conditions (5.4 and 7.4) were used just for MNP-F127-3-DOX due to its higher DLC percentage (Figure 5b). The final data revealed better DOX release under acidic environments, which can be accredited to the pH sensitivity of F127. Moreover, during the initial period, the release was faster in both environments, which may be due to the active compounds of DOX. Moreover, the results exposed the DOX-release ability of MNP-F127-3-DOX in a controlled manner under acidic environments, confirming the long-term delivery of DOX from the nanocomposites, which is a key factor for decreasing the side effects and increasing drug accumulation in tumor tissues. Thus, the release of DOX from the nanocomposites was triggered by pH, which decreased DOX loss throughout blood transport, thereby increasing the efficacy of the therapeutic drugs. To ensure the stability of the drug loaded in the MNP-F127-3-DOX nanocomposites, DOX-release data were measured using four different kinetic models, including the First-order, Korsmeyer–Peppas, Higuchi, and Weibull models, as displayed in Figure 6a–d. DOX molecules were chemically bound to the polymer on the surface of the MNP. The aforementioned kinetic models were fitted according to the drug release data obtained for the pH environment (pH = 5.4 and 7.4).

As shown in Figure 6, the First-order kinetics model reported lower R^2^ values, which demonstrated that the model was not the best fit for the experimental results. Nevertheless, a slight increase in the R^2^ values was attributed to the acidic environment, demonstrating pH-dependent release kinetics. After analysis, the Weibull and Higuchi models resulted in the best fit in terms of describing the DOX-release mechanism. These results indicate that DOX release occurred through a complex and anomalous mechanism, possibly due to the swelling behavior of the polymer [62,68]. Table 1 summarizes the correlation coefficients (R^2^) of each model for each pH condition.

### 3.3. In Vitro Cytotoxicity Studies

**Figure 7:**
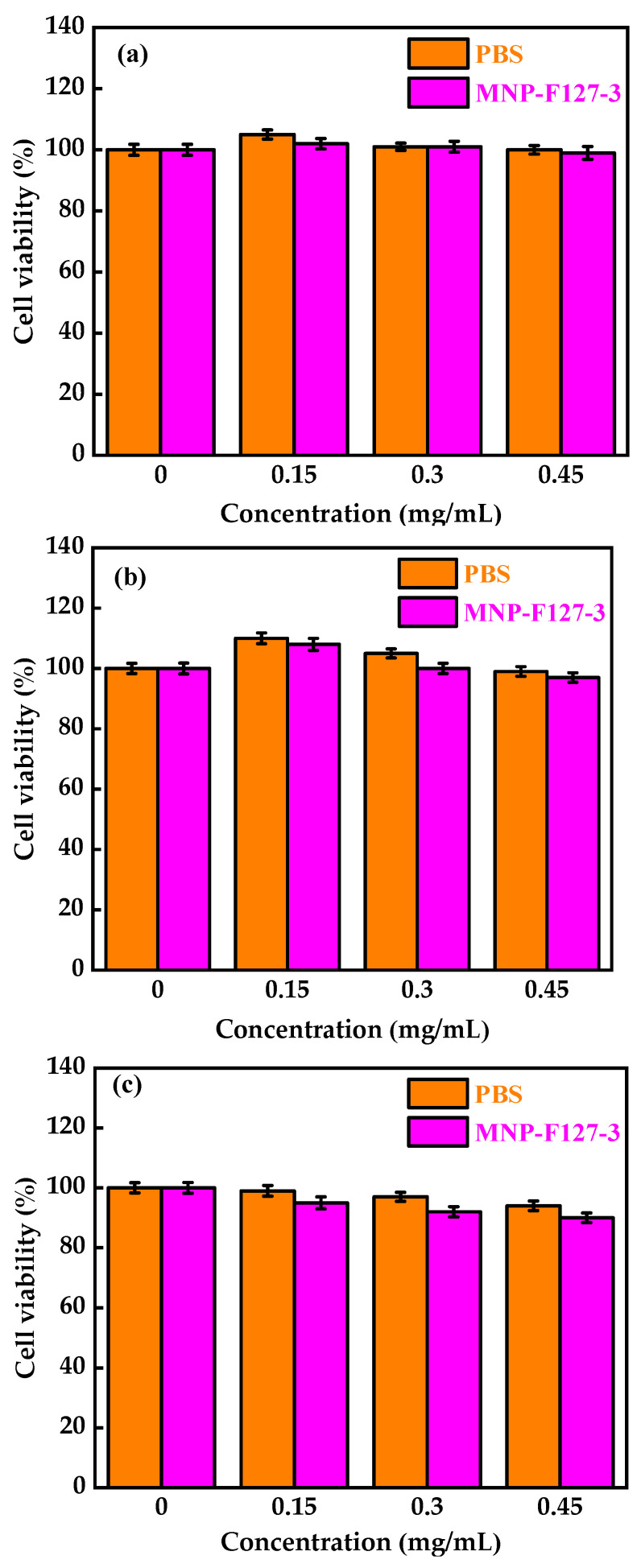
Cell viability of MNP-F127-3 and PBS over the HepG2 cell line at (**a**) 24, (**b**) 48, and (**c**) 72 h, respectively.

**Figure 8:**
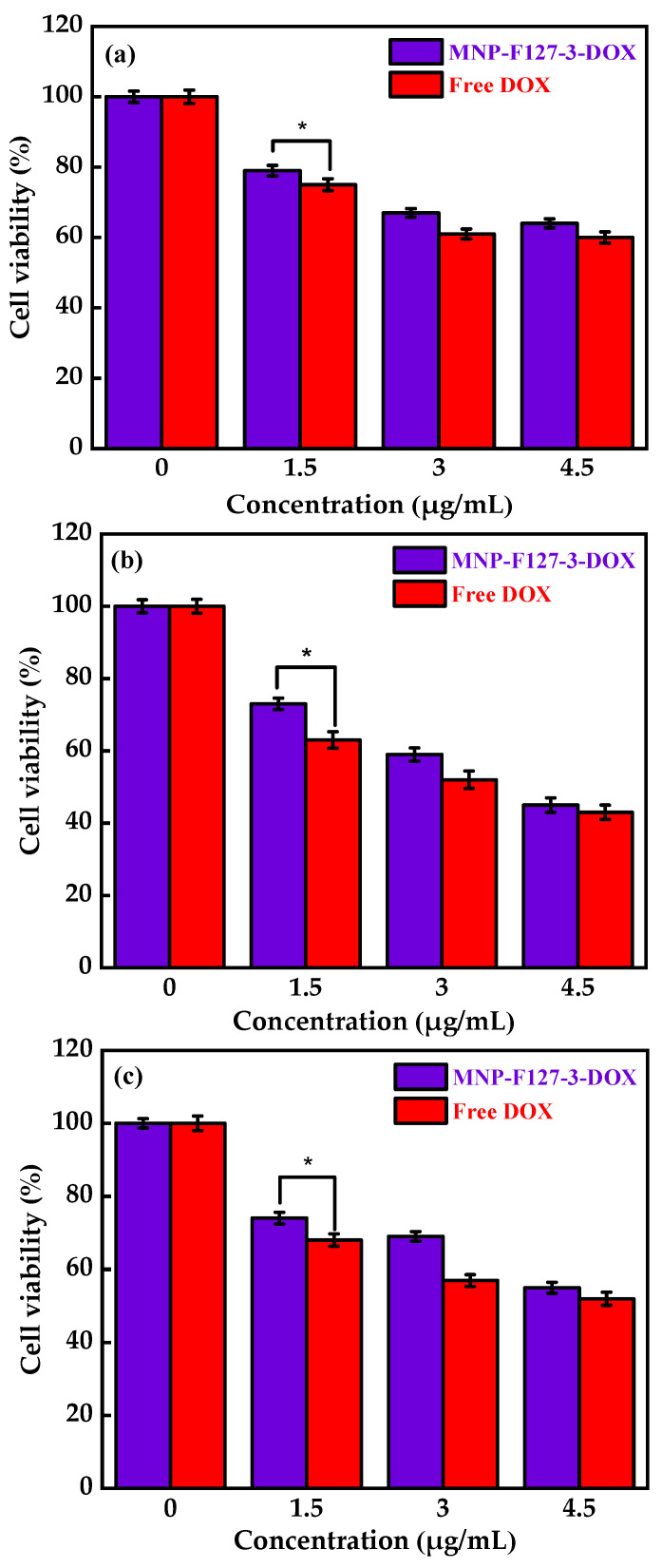
Cell viability of MNP-F127-3-DOX and free DOX over the HepG2 cell line at (**a**) 24, (**b**) 48, and (**c**) 72 h, respectively. * *p* < 0.05 is known to be statistically significant.

To evaluate the in vitro biocompatibility and pharmacological activity of the nanocomposites, an MTT assay was performed. The MNP-F127-3-DOX nanoparticles were selected for this analysis due to their high DLC and DLE (65 ± 0.12 and 13 ± 0.79%, respectively). The assay was carried out using increasing concentrations of control, PBS, MNP-F127, MNP-F127-DOX, and free DOX. As revealed in Figure 7a,b, cancerous HepG2 cells were exposed to different concentrations of PBS and MNP-F127-3. The final data showed nontoxic effects after 24, 48, and 72 h for both samples. Furthermore, HepG2 cells treated with MNP-F127-3-DOX and free DOX showed a survival rate of approximately 60–50%, suggesting effective cell growth inhibition (Figure 8a,b). The data showed significant concentration- and time-dependent cell growth inhibition for MNP-F127-3-DOX and free DOX.

### 3.4. Cellular Uptake

**Figure 9:**
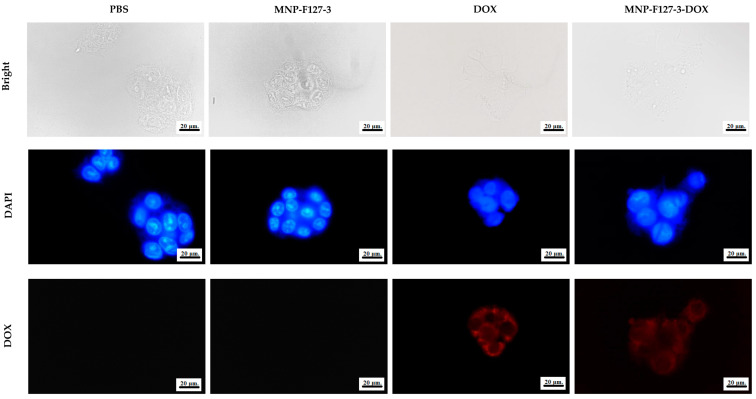
Cellular uptake of HepG2 cell lines treated with PBS, MNP-F127-3, free DOX, and MNP-F127-3-DOX.

To further confirm that the effect on cell viability was caused by apoptosis induced by DOX delivered into the cytoplasm, fluorescence microscopy was performed. The cellular uptake of PBS, MNP-F127-3, MNP-F127-3-DOX, and free DOX was analyzed in the HepG2 hepatocellular cancer cell line. As shown in Figure 9, the fluorescence microscopy images showed that cells treated with MNP-F127-3-DOX and free DOX exhibited red fluorescence, which confirmed successful uptake by the cell line. Additionally, fluorescence within the nuclei was significantly reduced, indicating that MNP-F127-3-DOX carried the anticancer drug DOX to the cells efficiently, thereby suggesting successful internalization of the nanoparticles by the cells. In contrast, cells treated with PBS and MNP-F127-3 did not show red fluorescence, demonstrating that the samples did not carry DOX. In addition, as shown in Figure 9, cells treated with PBS and MNP-F127-3 showed relatively uniform fluorescence distribution in the cytoplasmic compartment. Moreover, no noticeable morphological irregularities in the cells were detected, suggesting that the nanocomposites were well tolerated by the HepG2 cell lines. The uptake of nanoparticles by cellular systems is mediated by a process known as endocytosis and its stimulation is typically attributed to the physicochemical characteristics and properties of the nanocomposites, such as size, shape, and surface chemistry [67,68]. Nanocomposites can enter living cells frequently via many endocytic routes. Nevertheless, passive penetration of the plasma membrane can occur as a substitute. Upon endocytosis, the nanocomposites are surrounded first by endocytic vesicles and are consequently not directly transported to the cytosol. On the contrary, nanocomposites internalized via membrane permeation are transferred directly into the cytoplasm, which could be the better route, particularly for targeted drug delivery [69,70,71].

## 4. Conclusions

A novel magnetic drug delivery system was developed in which a layer of F127 was coated onto DOX-conjugated MNPs for liver cancer treatment. The obtained results demonstrated that the nanocomposites were properly synthetized, functionalized, and selectively internalized in HepG2 cells and induced cellular inhibition. The drug release demonstrated anticancer activity under acidic pH environments, releasing DOX in a controlled manner, which confirmed the pH sensitivity of the nanocomposites. The MTT assay demonstrated no cytotoxicity in HepG2 cells treated with MNP-F127, suggesting a survival rate higher than 90%. In contrast, cells treated with MNP-F127-3-DOX and free DOX displayed significant cellular inhibition. Additionally, fluorescence microscopy images showed successful drug internalization into cancer cells, mediating cellular apoptosis in cells treated with MNP-F127-DOX. The results demonstrate that the synthesized smart nanocomposites can be used in drug delivery as nanocargoes for targeted hepatocellular cancer treatment.

## Figures and Tables

**Table 1:** Kinetics models for DOX release from MNP-F127-3-DOX nanocomposites at different pH values at 37 °C.

Kinetics Model	Parameter	pH 5.4	pH 7.4
^a^ First-order	R^2^	0.786	0.897
^b^ Korsmeyer–Peppas	R^2^	0.971	0.966
^c^ Higuchi	R^2^	0.979	0.938
^d^ Weibull	R^2^	0.981	0.960

Note: R^2^ value denotes the correlation coefficient. ^a^ First-order: A constant proportion of the drug is eliminated per unit time. ^b^ Korsmeyer–Peppas: Both diffusion- and erosion-controlled rate releases. ^c^ Higuchi: The drug release occurs only by diffusion. ^d^ Weibull: Release profile of matrix-type (e.g., polymer) drug delivery.

## Data Availability

Data will be made available upon request from the corresponding author.
